# Control strategies against COVID-19 in China: Significance of effective testing in the long run

**DOI:** 10.1371/journal.pone.0253901

**Published:** 2021-07-09

**Authors:** Yatang Lin, Fangyuan Peng

**Affiliations:** 1 Division of Public Policy and Division of Social Science, Department of Economics, Hong Kong University of Science and Technology, Clear Water Bay, Hong Kong; 2 Society Hub, Hong Kong University of Science and Technology, Clear Water Bay, Hong Kong; Xiamen University, CHINA

## Abstract

The COVID-19 pandemic has become a long-term crisis that calls for long-term solutions. We combined an augmented SEIR simulation model with real-time human mobility data to decompose the effects of lockdown, travel bans and effective testing measures in the curtailment of COVID-19 spread in China over different time horizons. Our analysis reveals that the significant growth in the detection rate of infectious cases, thanks to the expansion in testing efficiency, were as effective as city lockdowns in explaining the reduction in new infections up to mid-March. However, as we extended the analysis to July, increasing the detection rate to at least 50% is the only reliable way to bring the spread under control.

## Introduction

Up to January 31, 2021, more than 100 million cases of COVID-19 had been reported, with more than two million deaths. As the WHO declared COVID-19 a pandemic on March 11, 2020, the world is preparing to live with COVID-19 as a new normal. All-encompassing lockdowns and travel bans would wreck the global economy in the long run. As a result, countries are looking for middle-ground solutions that would neither dry out national medical resources nor paralyze the economy. As the first country to sustain a major COVID-19 shock in late December 2019, China has taken unprecedented countermeasures to contain the spread of disease, including physical distancing, travel bans, testing, case identification, and quarantine. The measures appear to be effective: after March 15, 2020, without considering the imported cases from other countries, there were less than 100 new cases reported per day in China. On April 8, China lifted its 76-day lockdown of Wuhan. The country was reopening businesses and schools gradually.

Drawing lessons from China’s COVID-19 experiences can be essential for policy-makers to take effective measures to stop the epidemic from continuing indefinitely. Previous research highlight the effectiveness of social distancing interventions and discuss about the the need for lockdowns [1, 2]. Our study aimed to quantitatively evaluate the contribution of three types of policies in the successful containment of COVID-19: city lockdown that aims at reducing within-city contact, cross-city travel restrictions, and an effective way of testing and isolating infected persons. Most importantly, we followed the full trajectory of COVID-19 development in China from January 10, two weeks before the drastic lockdown of Wuhan on January 23, to March 15, when the pandemic was contained. The unusually long period study compared to previous papers enabled us to *estimate* rather than simulate the policy effects.

In this paper, we evaluated the effectiveness of different containment strategies in halting the pandemic spread in both short- and long-term. We combined a networked metapopulation SEIR model featuring undocumented infections, actual mobility data, and Bayesian inference to simulate the counterfactual outbreak scenarios removing each one or a combination of the following three policies in place: i) city lockdowns, ii) intercity travel bans, and iii) testing, detection, and quarantine. Our estimates revealed that 11.4% [95% credible interval (CI): 9.7–13.0%] of the infected cases were unidentified before January 23, 2020. The rate grew to 92.5% [95% credible interval (CI): 85.9–94.5%] in early March, thanks to the boost in coronavirus testing capacity. We show that increasing the detection rate of infections from 11.4% to 92.5% alone would explain 75% of the reduction in infections from a no-policy baseline by March 15, 2020.

The most pronounced finding is that city lockdown appeared to be the more effective intervention in the short-run but effective testing, detection, and quarantine measures are essential in containing the COVID spread in the long run. By March 15, restoring within-city personal contact to its 2019 level would lead to a 678% growth in infections with all the other interventions unaffected, and removing intercity travel restrictions and effective testing treatment respectively would lead to a 3% and 477% growth in infected cases. As we extend the analysis to July, the counterfactual increase in infections would become 581%, 3% and **3.05×10^5^%** had the three classes of interventions lifted individually. In addition to this, we found that increasing the detection rate to at least 50% is the only sure way to bring the spread under control. Our work highlights the necessity of growing testing efficiency in combating the current and future public health pandemics.

Perhaps the strongest policy implication that emerges from our evidence is that the detection rate of infectious cases has to be higher than 50% to bring the pandemic under control in the presence of strict city lockdown at the beginning of the outbreak, and higher than 70% without. [3] estimated the detection rate of COVID-19 infections across 10 countries based on a demographic scaling model and age-specific infection fatality rates (IFRs). By mid-May, the estimated detection rate was less than 20% in Italy, 45% for the U.S., and 55% for Germany, which could explain the diverging performance in COVID-19 control across these countries. Consequently, investments in testing capacity and contact-tracing systems should be placed in a high priority to prevent ongoing secondary outbreaks of COVID-19 or similar future outbreaks of other emergent infectious diseases.

## Materials and methods

### Data

#### Observations of confirmed COVID-19 cases

We have compiled a city-level health outcome dataset in China for 339 cities from January 10, 2020, to March 15, 2020. From January 24, 2020, onwards, data are obtained from the public dataset Ding Xiang Yuan (DXY) that reports daily statistics across Chinese cities (Source: https://ncov.dxy.cn/ncovh5/view/pneumonia). We used a web scraper program to obtain data from DXY 2–4 times every day. Data before January 24 can be obtained from the official website or official Weibo of National and Provincial Health Commissions in China.

#### Intercity mobility

We obtain inter-city population migration data from Baidu Migration (Source: http://qianxi.baidu.com/), a travel map offered by the largest Chinese search engine, Baidu. They calculate population flow based on the Baidu mapping app user’s location and show the trajectory and characteristics of the population migration on the platform. For each of the 365 Chinese cities, the Baidu migration data reports the population inflow from the top 100 origin cities and outflow to the top 100 destination cities between January 21 and March 23 in 2019, and between January 10 and March 15 in 2020. In our main analysis, we rely on inter-city migration in 2019 to simulate the counterfactual spatial transmission of COVID-19 without traffic bans. Naturally, the reduction in intercity mobility in 2020 from its 2019 level is a combination of policy effects and individuals’ voluntary avoidance behavior as a result of increased awareness. Our analysis is going to capture the composite impact of these two channels.

#### Within-city mobility

Apart from the intercity data, Baidu also provides the daily within-city mobility data for each city in the sample period from a separate data product. The data is generated based on Baidu Map app usage within a city. We rely on this data to describe within-city mobility. Since Baidu’s app may not cover all population, we compare it to the mobility data in [4], which used nationwide mobile phone data to track population outflow from Wuhan from January 1 to January 24. S7 Fig presents the comparison. The population mobility measured by mobile phone data sources is 5.5–6.5 times of the Baidu index. Therefore, We multiply inter-city mobility measures from Baidu by the multiplication factor of 6.

### Model framework

**Fig 1:**
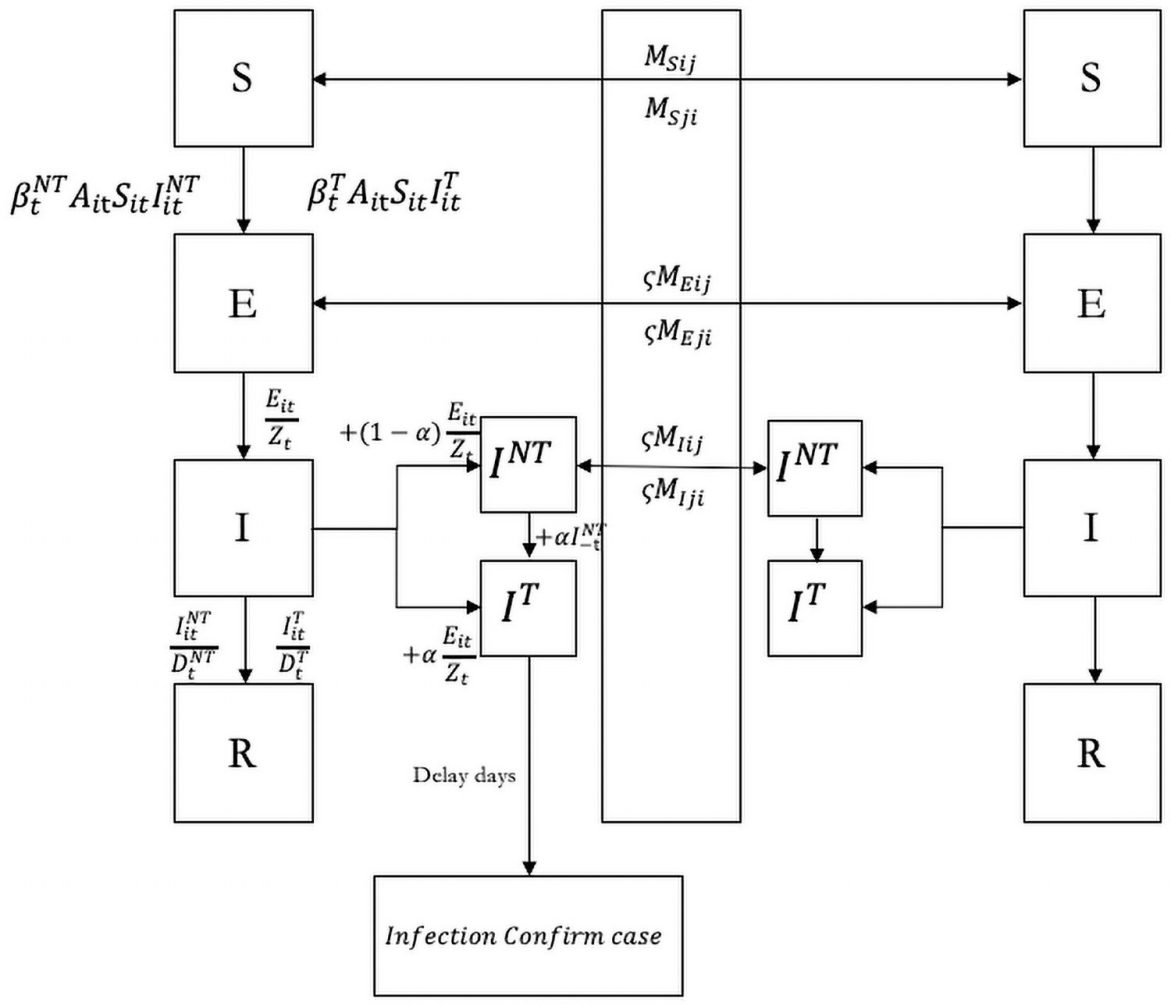
SEIR model. Graphical scheme representing the interactions among different stages of infection in SEIR Model. In this model, *S*_*it*_, *E*_*it*_, $I_{it}^{T}$, $I_{it}^{NT}$ and *N*_*it*_ denote the susceptible, exposed, detected infected, undetected infected and total population in city *i* and time *t*.$\beta_{t}^{NT}$ and $\beta_{t}^{T}$ are the rate of transmission for undetected infected individuals and detected infected individuals, respectively. *α*_*t*_ is the testing rate in time t.*Z*_*t*_ denotes latency period through which patients switch from exposed stage to infection stage. *A*_*it*_ is within-city population flow in city i in time t. Spatial spread of the disease is governed by the daily number of people travelling from city j to city i in time t(*M*_*ijt*_).

We modeled the transmission of COVID-19 using a Susceptible-Exposed-Infected-Recovered (SEIR) framework that can flexibly generate patterns of spatial transmission (See Fig 1 for an illustration of the model structure). Our model adapts from [5]. Similar models have been developed to study the spread of disease in Spain and Italy, such as [1, 2].

The transmission model adopts the following metapopulation structure:

$$\begin{array}{c} S_{it+1}=S_{it}-\frac{b_{t}^{T}A_{it}S_{it}I_{it}^{T}}{N_{i}}-\frac{b_{t}^{NT}A_{it}S_{t}I_{it}^{NT}}{N_{i}}-\sum_{j}\frac{M_{jit}S_{jt}}{N_{j}-I_{jt}^{T}}+\sum_{j}\frac{M_{ijt}S_{it}}{N_{i}-I_{it}^{T}} \end{array}$$ (1)

$$\begin{array}{c} E_{it+1}=E_{it}+\frac{b_{t}^{T}A_{it}S_{it}I_{it}^{T}}{N_{i}}+\frac{b_{t}^{NT}A_{it}S_{t}I_{it}^{NT}}{N_{i}}-\frac{E_{it}}{Z_{t}}-\sum_{j}\frac{M_{jit}\zeta_{t}E_{jt}}{N_{j}-I_{jt}^{T}}+\sum_{j}\frac{M_{ijt}\zeta_{t}E_{it}}{N_{i}-I_{it}^{T}} \end{array}$$ (2)

$$\begin{array}{c} I_{it+1}^{T}=I_{it}^{T}+\alpha_{t}\frac{E_{it}}{Z_{t}}-\frac{I_{it}^{T}}{D_{it}^{T}}+\alpha_{t}I_{it}^{NT} \end{array}$$ (3)

$$\begin{array}{c} I_{it+1}^{NT}=I_{it}^{NT}+\left(1-\alpha_{t}\right)\frac{E_{it}}{Z_{t}}-\frac{I_{it}^{NT}}{D^{NT}}-\alpha_{t}I_{it}^{NT}-\sum_{j}\frac{M_{jit}\zeta_{t}I_{jt}^{NT}}{N_{j}-I_{jt}^{T}}+\sum_{j}\frac{M_{ijt}\zeta_{t}I_{it}^{NT}}{N_{i}-I_{it}^{T}} \end{array}$$ (4)

$$\begin{array}{c} N_{it+1}=N_{it}-\sum_{j}M_{ji}+\sum_{j}M_{ij} \end{array}$$ (5)

where *S*_*it*_, *E*_*it*_, $I_{it}^{T}$, $I_{it}^{NT}$ and *N*_*it*_ are the susceptible, exposed, detected infected, undetected infected and total population in city *i* and time *t*. The interactions among different stages of infection are visually represented in Fig 1. The parameters are defined as follows:

-
$b_{t}^{NT}$ demotes the probability of transmission among detected infected persons. *A*_*it*_ denotes within-city population mobility. Their product is going to be the rate of transmission for undetected infected individuals($\beta_{it}^{NT}$). $b_{t}^{T}$ is the probability of transmission for undetected infected individuals. Typically $b_{t}^{T}$ should be smaller than $b_{t}^{NT}$, provided that confirmed infected persons are properly quarantined.
- *α*_*t*_ is the testing rate in time t, which is defined as the ratio between the number of documented infections in time t and the sum of cumulated undetected patients carried over from last period and new patients in time t.$\alpha_{t}=I_{it}^{T}/\left(I_{it}^{NT}+\frac{E_{it}}{Z}\right)$
- *Z*_*t*_ denotes the latency period through which patients switch from exposed stage to infection stage. *D*_*t*_ is the infectious period that patients could infect the susceptible population. $D_{t}^{T}$ is the infectious duration for detected infections while $D_{t}^{NT}$ is for undetected infections. *D*_*t*_ is typically lower than $D_{t}^{NT}$ provided that detected infected individuals would be properly treated and become well sooner.
- *A*_*it*_ is within-city population flow in city i in time t. The spatial spread of the disease is governed by the daily number of people traveling from city j to city i in time t(*M*_*ijt*_). To capture the fact that exposed and undetected individuals might travel less to other cities, likely because of family members’ illness or voluntary avoidance behavior following the reports of epidemic hot spots, we add a multiplicative factor, *ζ* smaller than one. We further assume that individuals in the tested group who have been admitted by local hospitals do not move between cities.

In this model, the effective reproduction number (*R*_0_) is calculated as $R_{0}=\alpha_{t}\beta_{t}^{T}A_{t}D_{t}^{T}+\left(1-\alpha_{t}\right)\beta_{t}^{NT}A_{t}D_{t}^{NT}$.

### Estimation and simulation

We infer model epidemiological parameters using an iterated filtering (IF) approach [5]. In our model, we consider the unreported infection may be tested later, so we add $\alpha_{t}I_{it}^{NT}$ source code. We divided the full sample from January 1 to March 15 into five subperiods: January 10 to January 23, January 24 to February 2, February 3, February 12, February 13 to February 22, and February 23 to March 15. We estimated the key parameters (*α*, *β*_*documented*_, *β*_*undocumented*_, *γ*_*documented*_, *γ*_*undocumented*_) for each period. The first period spans from the first day of Spring Festival to the day of Wuhan lockdown; the following three periods cover each of a 10-day interval up to February 22. After February 22, daily new cases dropped to a new level, and most of the containment policies were relaxing, so we set February 22 to March 15 as the final period.

Similar to [5]’s algorithm, the core model structure (Eqs 1–5) was integrated stochastically using a 4th order Runge-Kutta (RK4) scheme. Specifically, for each step of the RK4 scheme, each unique term on the right-hand side of Eqs 1–4 was determined using a random sample from a Poisson distribution. The initial values of the parameters were drawn using Latin hypercube sampling from uniform distributions with pre-specified ranges. The initial range for $\beta_{t}^{NT}$ was set as $0.22\leq \beta_{t}^{NT}\leq 0.45$ which is based on transmission rate range (i.e. [0.8, 1.5] /ref) and mean value of within city flow 3.5. $\beta_{t}^{T}$ is equal to the transmission possibility of undetected infected patients multiply by a multiplicative factor $\beta_{t}^{T}=\mu_{\beta }\times \beta_{t}^{NT}$. The initial ranges for *α*_*t*_, *μ*_*β*_ and $\zeta_{t}^{T}$ were chosen to cover most possible values, i.e. [0, 1]. The initial ranges for the latency *Z*_*t*_ were set from /ref ($2\leq Z_{t}^{T}\leq 5$). The initial ranges for the infection periods D in /ref is set as [2, 5], since detection and treatment of infected patients may be reduce their infectious period, so we extend the range of the average duration of infection D to 1–5 days, where The average duration of infection for tested infected patients $D_{t}^{T}$ is 1–3 days and the average duration of infection for untested infected patients $D_{t}^{NT}$ equal to 3–5 days.

Cities in Hubei published confirm case including clinical confirm cases from 2.13, which counted patients who met clinical criteria through chest imaging and may not have had epidemiological links or a positive PCR test Confirmed cases in Wuhan was more than 12 times higher than that in the previous day, so the reported rate in Wuhan may be different from other cities and we create a multiplication factor *μ*_*Wuhan*_ for Wuhan and testing rate in Wuhan equal to *μ*_*Wuhan*_×*α*. To simulate the sharp increase in cities in Hubei, we assume a testing rate of cities in Hubei equal to 1 on February 13. The initial exposed population *E*_*wuhan*_ and initial undocumented infected population, *I*_*wuhan*_ are set from a uniform distribution [0, *Seed*_*max*_]. *Seed*_*max*_ is estimated at [1000, 4000] in January 10 [5], and we compare the fitting results under different initial values, and found that *Seed*_*max*_ = 3000 is the best fitting value. (See S4 Fig). We set the initial exposed population and initial undocumented infected population of other cities based on the number of travelers from Wuhan to the city i on the first day of Chunyun. (*E*_*i*_ = *M*_*iwuhan*_ *E*_*wuhan*_/∑_*i*_ *M*_*wuhan*_ and $I_{i}^{NT}=M_{iwuhan}I_{wuhan}^{NT}/\sum_{i}M_{iwuhan}$, *M*_*iwuhan*_ means the number of travelers from Wuhan to city i) In our model, we also consider a reported delay for tested infection. Cases are classified as suspected before reported officially as confirmed cases, before that they must be tested at least two times. Suspected cases are sent to designated hospitals and quarantined before official confirmation. Details on the reported delays could be found in Diagnosis and Treatment Protocol for COVID-19, which published by the National Health Commission of the People’s Republic of China. Source: http://www.gov.cn/zhengce/zhengceku/2020-02/05/5474791/files/de44557832ad4be1929091dcbcfca891.pdf. Therefore, reported delay refers to the time interval between a person was admitted by a hospital and the observed confirmation of that individual infected case. In reality, many cases were confirmed after multiple tests and the supply of testing reagent was insufficient at the beginning of the pandemic. Since the reported delay in our research is different from that in [5], we re-calculate gamma distribution parameters based on our reported delay definition. To estimate this delay period, we examined panel data on some confirmed patients. Reporting Delay is calculated by a real-time database of the individual-level epidemiological dataset. Data Source: https://github.com/beoutbreakprepared/nCoV2019. The dataset records geocoded COVID cases with extra information on symptoms, key dates (date of onset, admission, and confirmation), and the travel history of patients. We calculate the delay period as the duration between date of onset and date of confirmation. The distribution of reported delay is shown in S6 Fig. Through the event study (S5 Fig), we found that the delay had a high improvement after February. Therefore, we simulated the confirmed delay before and after 2.1. We found that the time interval could be fit by the Gamma distribution(a = 3.55, b = 1.22, LL = 1669.71) before 2.1 and a = 3.86, b = 0.78, LL = 522.82 after 2.1) (S6 Fig). Since Hubei province includes clinical diagnosis to confirm patient, we set reported delay in Hubei equal to 0 in 2.12.

We trace the spatial spread of COVID-19 across cities with mobility data from Baidu Migration, a data service provided by the largest Chinese search engine. Baidu collects the population mobility information from real-time location records of smartphones that use its mapping app. The platform reports a bilateral migration index between 36057 city pairs per day for 365 Chinese cities between January 12 and March 26 in 2019, and between January 1 and March 15 in 2020. It also publishes daily within-city mobility data for each city during the sample period. The period covers the annual “Chunyun” (Spring Festival travel season) mass migration cycle. To derive a counterfactual scenario had the restrictions on inner and inter-city mobility never been implemented, we align the 2019 and 2020 Baidu mobility data on the basis of relative timing to the Spring Festival. For example, we assume that without intercity travel bans, the counterfactual number of travelers between city pairs on January 23, 2020 (2 days before Spring Festival) will be the same as the observed number of travelers on February 3, 2019. Similarly, the inner-city mobility reduction from the 2019 baseline level was used to estimate the effects of city lockdown on contact reductions.

To evaluate the role of testing, detection, and post-diagnosis quarantine, we divided infections into documented and undocumented cases (*I*^*T*^ and *I*^*NT*^ in Fig 1). The two types of infections have different rates of transmission (*β*) and infectious period (1/λ). In light of the model, extensive testing and detection (higher detection rate *α*) help to reduce the transmission risk of infected persons as they get quickly isolated and treated. The individual behaviors and policy intervention may influences the epidemic evolution [6]. To capture the changes in epidemiological characteristics of the outbreak over time, especially after January 23, when serious control measures were implemented, and after Feb 08, when industries were gradually reopened, we divided the full sample from January 1 and March 15 into five subperiods: January 10 to January 23, January 24 to February 2, February 3 to February 12, February 13 to February 22, and February 23 to March 15. We estimate the key parameters (*α*, *β*_*documented*_, *β*_*undocumented*_, *γ*_*documented*_, *γ*_*undocumented*_) separately for each subperiod, and maps the changes to observed improvements in control measures in reality. To better characterize the overwhelmed testing capacity at the early outbreak in Wuhan, we allow the detection rate *α* to differ between Wuhan and the rest cities.

## Results and discussions

### Validation of the model-inference framework

**Fig 2:**
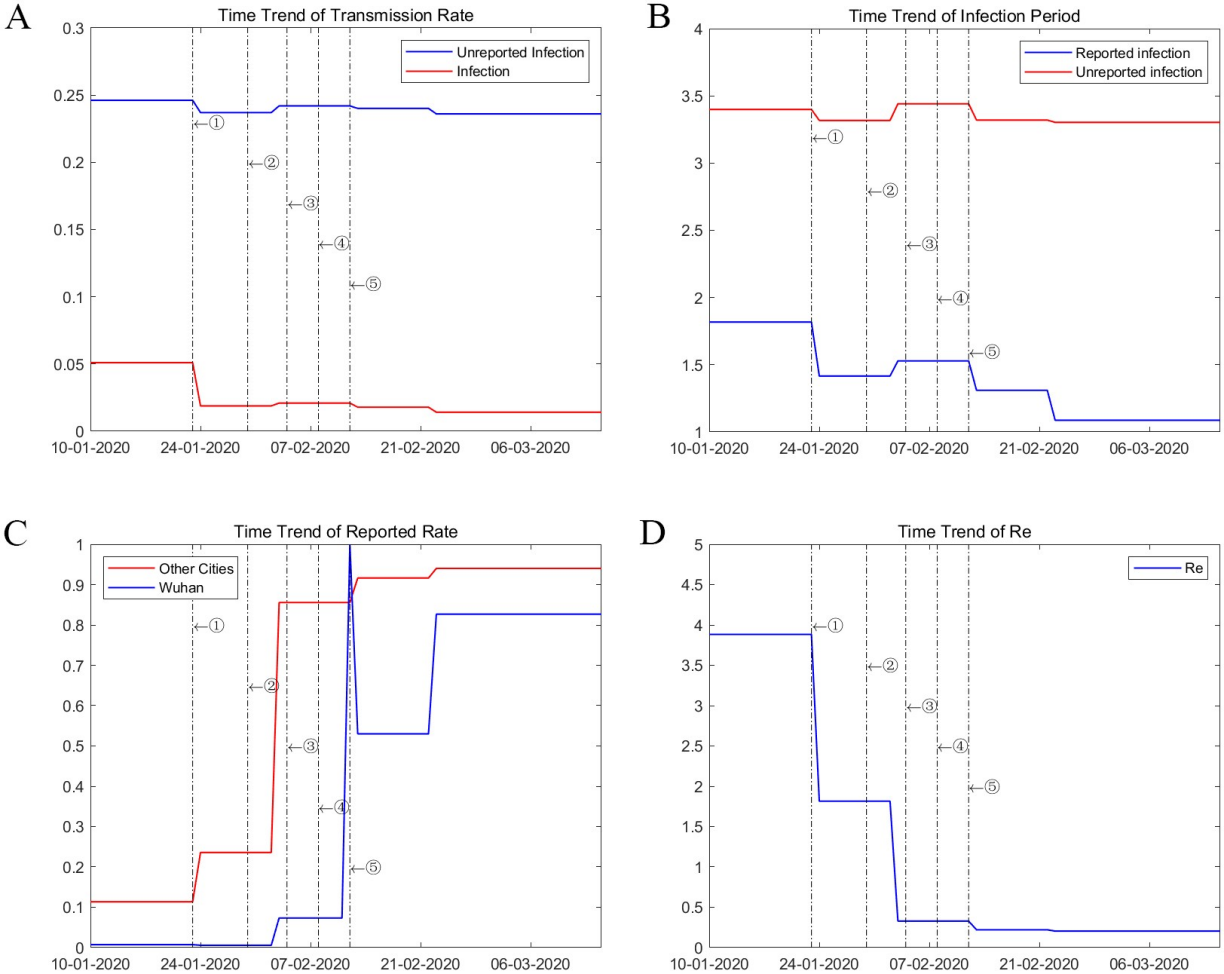
Time trend of parameters. These graph plot the changes in parameter value (A) Transmission Rate, (B)Infectious Period, (C)Detection Rate, and (D)Effective reproduction number estimated over five subperiods: January 10 to January 23, January 24 to February 2, February 3 to February 12, February 13 to February 22, and February 23 to March 15.

We estimated key parameters of the model using an iterated filter-ensemble adjustment Kalman filter (IF-EAKF) approach [5]. The framework identifies the maximum likelihood estimates of key parameters in Table 1. We estimated the model for five subperiods from January 10 to March 15, 2020, and mapped the changes in key parameters to observed changes in different non-pharmaceutical interventions in reality. The probability of transmission (*b*) for undocumented infections was less than 20% of that for documented cases from January 10 to January 23, 2020. The ratio further dropped to 12% after February 3. The infectious period for positive cases also dropped from 1.82 in late January to 1.09 in early March. Both effects could be attributable to improvements in the treatment capacity and practices in handling confirmed patients. A notable example is the introduction of Fangcang shelter hospitals, a rapidly-constructed and low-cost medical infrastructure that provided basic isolation, triage, medical care, monitoring, and referral services to clinically confirmed patients. The development of Fangcang hospitals starting from February 5, 2020, significantly reduced intrafamily transmission associated with home isolation and was considered a critical move in balancing the strained medical system in Hubei [7]. Meanwhile, the detection rate *α* proliferated from less than 1% in Wuhan before January 23 to more than 70% in early March, as shown in Fig 2. The detection rate for other cities grew steadily for other cities from 11.3% [95% credible interval (CI): 9.7–13.0%] at the onset of the pandemic to 92.5% [95% credible interval (CI): 85.9–94.5%] in early March, accompanied by a significant reduction in the transmission rate and infectious period of confirmed patients, evidence consistent with substantial improvement in testing and treatment capabilities. Our estimated basic reproductive number, R0, is 3.88 [95% credible interval (CI): 3.70–4.32], consistent with other recent estimates in similar settings [8–11].

**Table 1:** Best-fit model estimates for key epidemiological parameters.

Parameter	10 January–23 January	24 Jan–2 Feb	3 Feb– 12 Feb	13 Feb–22 Feb	23 Feb–15 Mar
Transmission Rate *β*^*T*^ (Reported)	0.05 (0.03,0.08)	0.02 (0.01,0.03)	0.02 (0.02,0.05)	0.02 (0.01,0.03)	0.01 (0.01,0.02)
Transmission Rate *β*^*NT*^ (Unreported)	0.25 (0.24,0.25)	0.24 (0.23,0.24)	0.24 (0.23,0.28)	0.24 (0.23,0.26)	0.24 (0.23,0.24)
Mobility Probability	0.1 (0.09,0.12)	0.92 (0.9,0.94)	0.73 (0.41,0.91)	0.69 (0.25,0.9)	0.49 (0.06,0.92)
Latent Period	4.57 (4.48,4.65)	4.67 (4.57,4.74)	4.41 (2.46,4.7)	3.7 (2.32,4.66)	3.79 (3.03,4.41)
Testing Rate	0.11 (0.1,0.13)	0.24 (0.13,0.68)	0.86 (0.24,0.94)	0.92 (0.12,0.95)	0.94 (0.86,0.95)
Infection Period (Reported)	1.82 (1.43,2.24)	1.42 (1.14,2.3)	1.53 (1.13,2.44)	1.31 (1.1,2.05)	1.09 (1.06,1.55)
Infection Period (Unreported)	3.4 (3.27,3.68)	3.32 (3.2,3.63)	3.44 (3.22,4.03)	3.32 (3.2,3.6)	3.3 (3.22,3.76)
Relative testing rate (Wuhan)	0.01 (0.01,0.01)	0.01 (0,0.04)	0.07 (0.01,0.09)	0.53 (0.05,0.68)	0.83 (0.56,0.87)

Notes: This table shows best-fit model posterior estimates of the median and 95% confidence intervals for key epidemiological parameters derived from 200 simulation of the model. To simulate the sharp increase in cities in Hubei, we assume a testing rate of cities in Hubei equal to 1 on February 13, so the testing rate in Hubei in February 13 is not estimated.

### Simulation results

We present simulations of reported cases generated by the model in Fig 1. The simulation matches well with the observed outbreaks for both Hubei province and the rest of China, even though we did not target at matching the two subgroups separately. The explicit modeling of reporting activities also makes our model more flexible to account for surges in reported cases as a result of changes in case identification criteria. Estimation of epidemiological models on China from previous papers ([12–14]) usually stops before February 13. Because on that day, China revised its case definition in Hubei, which counted patients who met clinical criteria even without a positive PCR test, purported to clear the backlog of COVID-19 tests. To account for this outlier, we manually set *α* = 1 for Wuhan on February 13, 2020.

**Fig 3:**
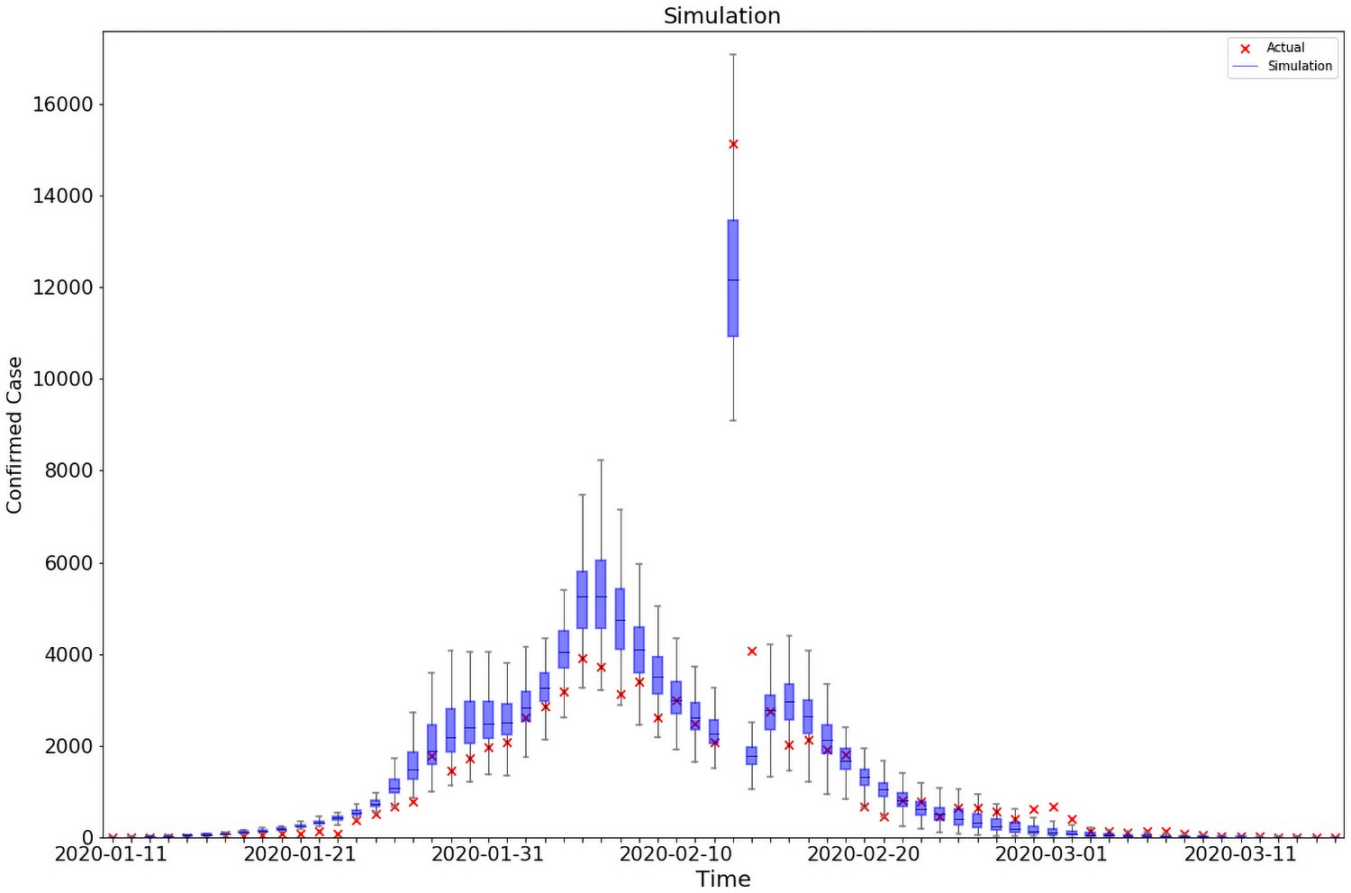
Best-fit model. This graph compares the daily official reported number of cases to the simulated ones from 200 simulations using the fully-estimated model from Jan 10,2020 to March 15,2020. The orange x deontes the number of daily reported cases. The blue box and whiskers show the median, interquartile range(IQR), and 1.5IQR derived from 200 simulations using the fitting model with parameters estimated from Table 1. *R*^2^ = 0.86–0.97.

**Fig 4:**
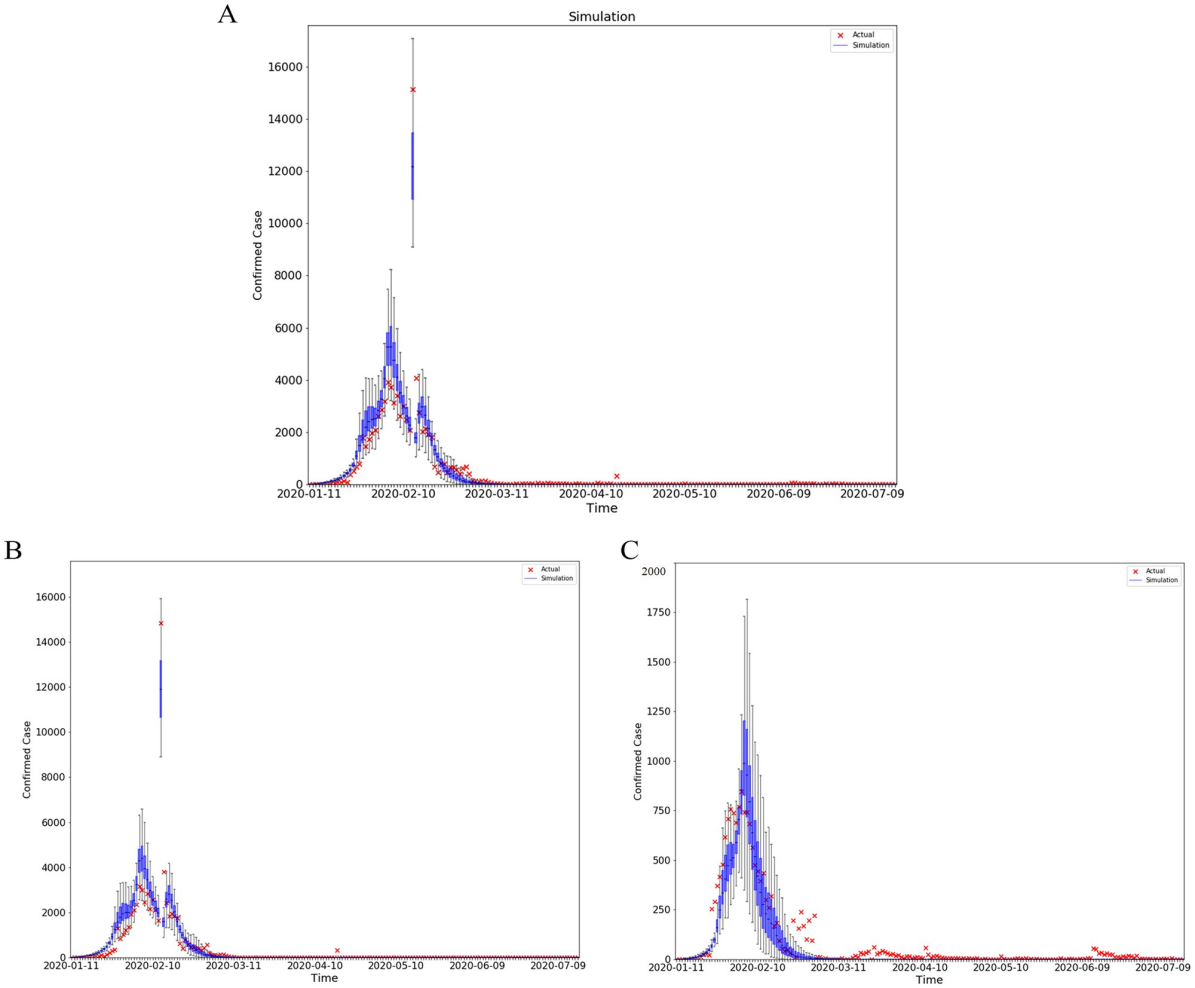
Prediction. These graphs compare the daily official reported number of cases to the simulated ones from 200 simulations using the fully-estimated model in all cities (A), Hubei province(AB), cities outside hubei province (C) from Jan 10,2020 to July 15,2020. The orange x deontes the number of daily reported cases. The blue box and whiskers show the median, interquartile range(IQR), and 1.5IQR derived from 200 simulations using the fitting model with parameters estimated from Fig 2. *R*^2^ = 0.86–0.97.

As is clear from Fig 3, the surge was well simulated in our model. This operation allows us to extend our analysis to March 15, the last day when Baidu mobility data were made available. An obvious benefit of extending the period of study is that we could look at changes in key parameters in response to new policy changes after February 13, including the opening of temporary hospitals and tentative re-opening of industries. Assuming that the control measures were kept at similar levels from March 15 to July 15, we borrow the parameter estimates and mobility measures from the last period of our sample (February 23 to March 15) to conduct an out-of-sample model validation exercise. As shown in Fig 4, the predicted cumulative infections on July 15 is only 17% higher than the actual ones, a sign that our model could capture the intensity of containment policies well.

Although we adopt the inference method in [5], we make some changes to their model, and thus the parameters (i.e. basic reproduction number *R*0) we infer may be inconsistent with their result. Firstly, definitions of the reported delay and testing rate are different in our research. Reported delay in our model refers to the time interval between a person admitted by the hospital and observational confirmation of that individual infection, which also can represent detection capability and reagent accuracy. Hence, the testing rate is defined as the number of patients admitted by a hospital during a fixed period of time divided by the number of untested infections during that period. In this way, the testing rate can reflect the acceptance capacity of the hospital. Their reported delay is the time interval between a person transitioning from latent to contagious and observational confirmation of that individual infection. Therefore, their detection rate means the probability that a new infection in a given day will be tested on the day or future. In addition, we not only consider the impact of inter-city population flow but also take into account the impact of within-city activities on disease transmission. Within-city population activities data from Baidu enable us to quantify the effect of policies that reducing the inner city (i.e. Lockdown). Specifically, we set within city population mobility as the people exposed to each day by infected people. In our model transmission rate equal to the product of the people exposed to each day by infected people (*A*_*it*_) and the probability of transmission (*b*_*it*_) when exposed (i.e. *β*_*it*_ = *A*_*it*_×*b*_*it*_). Population flow inter or inner cities and differences in parameters between detected and undetected infections allow us are taken into account in our model, allowing us to decompose the effect of different policies in controlling pandemic and to identify the most efficient combinations of policies.

**Fig 5:**
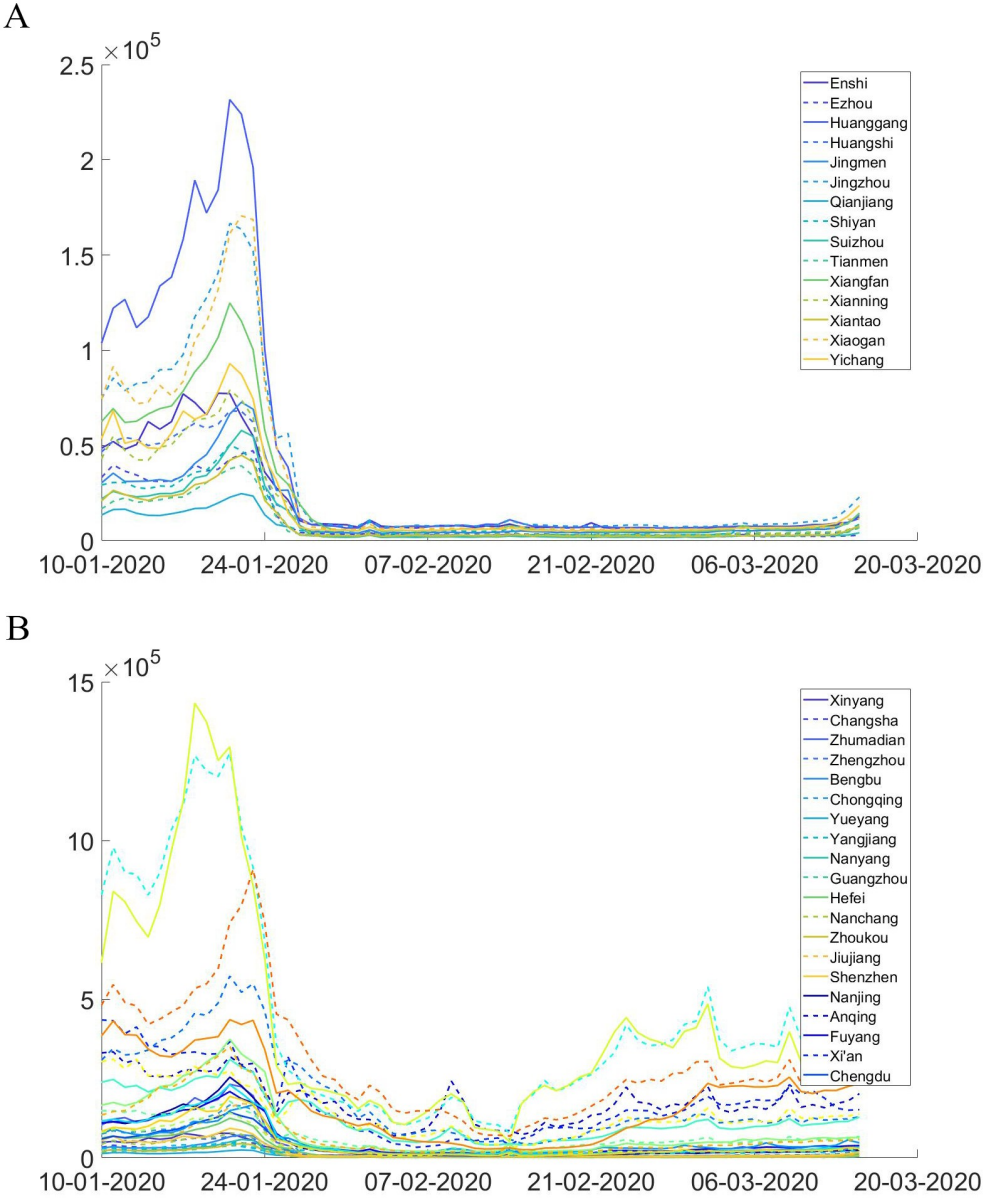
Inter-city population flows. These graphs plot the size of daily aggregate population inflow into cities in Hubei(a) and selected cities out of Hubei (b).

**Fig 6:**
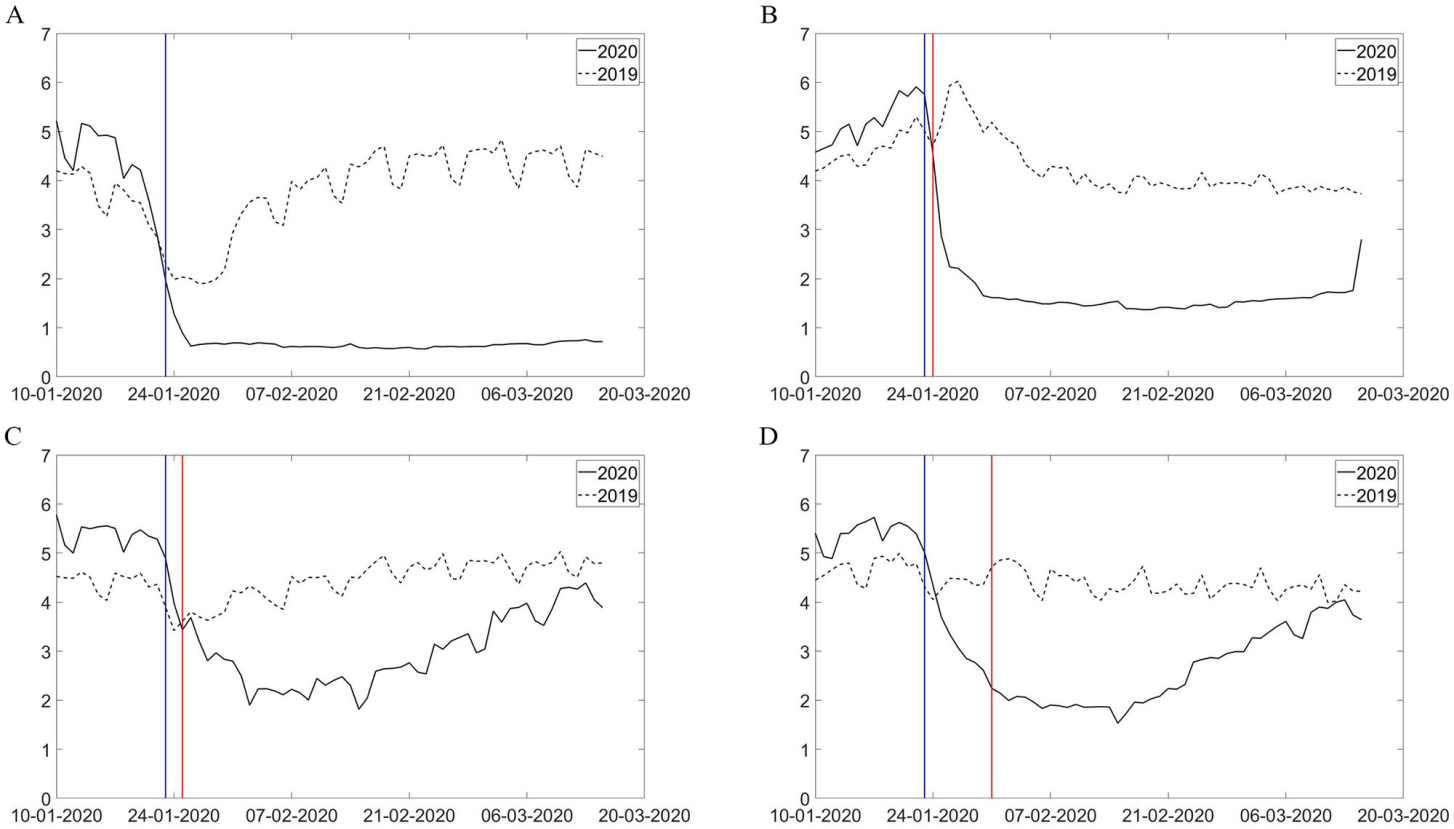
Within-city mobility. These graphs plot daily variations in within-city mobility in Wuhan(a), Huanggang(b), Qinghuangdao(c) and Chongqing(d) in 2019 and 2020. Blue Line indicates the date of Wuhan lockdown (Jan 23,2020) and red line indicates the date of each city’s own lockdown.

The impacts of city lockdown and intercity travel bans on mobility are captured by the reduction in within- and cross-city mobility indices in 2020 relative to their 2019 level, reported by Baidu Migration, a travel map offered by the largest Chinese search engine. A first glance at the real-time mobility data in Figs 5 and 6 showed significant reductions in population inflow into Hubei cities after January 21, and the trend never recovered up to mid-March. The within-city mobility is similar, with a significant drop after lockdown. To quantificationally evaluate the effect of these policies, we derive a counterfactual scenario had the restrictions on inner and inter-city mobility never been implemented by aligning the 2019 and 2020 Baidu mobility data on the basis of relative timing to the Spring Festival. For example, we assume that without intercity travel bans, the counterfactual number of travelers between city pairs on January 23, 2020 (2 days before Spring Festival) will be the same as the observed number of travelers on February 3, 2019. Similarly, the inner-city mobility reduction from the 2019 baseline level was used to estimate the effects of city lockdown on contact reductions.

**Fig 7:**
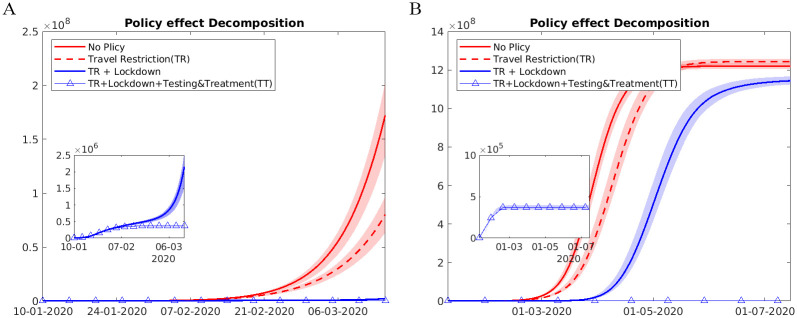
Alternative scenarios: Lifting combinations of different policies. This figure plots the trends in observed and counterfactual number of cumulative infections under several alternative scenarios from (A) Jan 1,2020 to Mar 15,2020, and from (B) Jan 1 to July 15. Data are presented as the median (solid line) and IQR (shading) of estimates (200 simulations). “No Policy” denotes the scenario in which no anti-contagion policies have been put in place. “Travel Restriction” denotes the case where only intercity travel bans were implemented after January 23. “TR+Lockdown” denotes the scenario in which both intercity travel restrictions and city lockdown were implemented. “TR+Lockdown+Testing&Treatment(TT)” denotes the baseline scenario in which all three policies have been implemented from January 23 to March 15.

**Fig 8:**
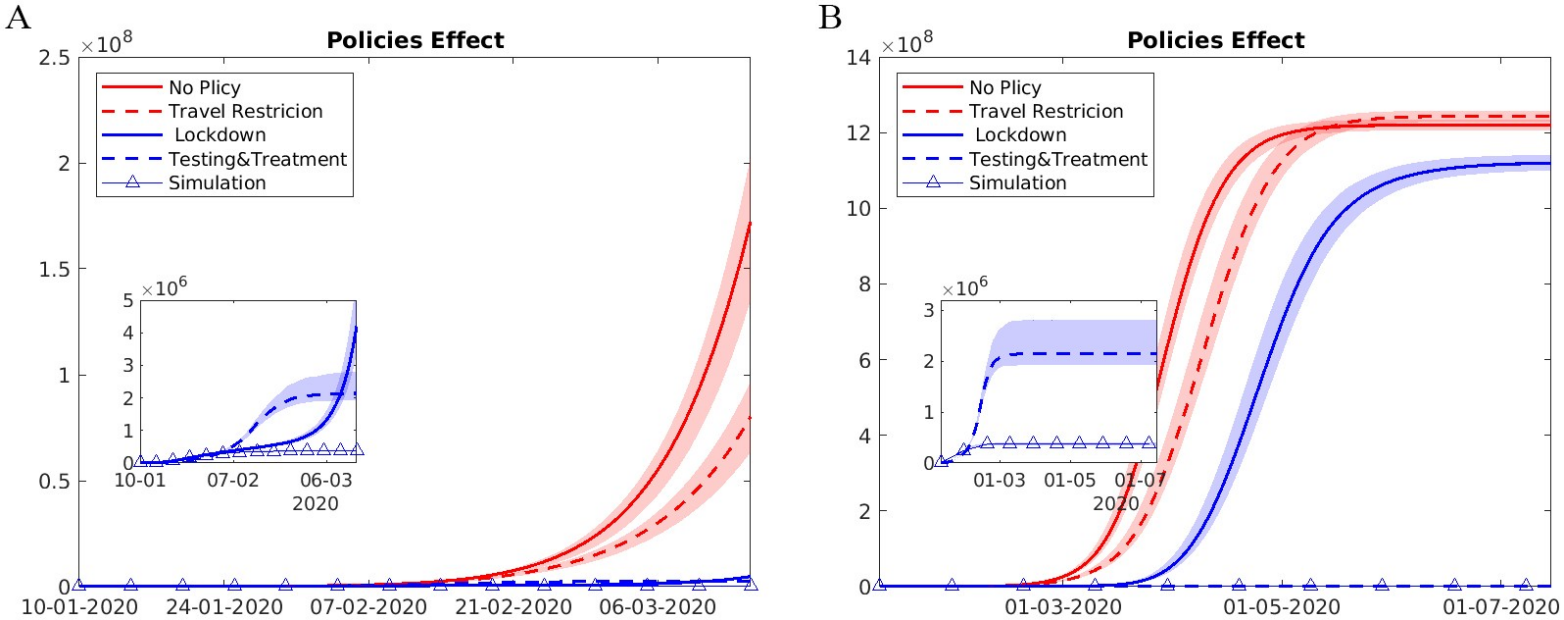
Alternative scenarios: Lifting each policy at once. This figure plots the trends in observed and counterfactual number of cumulative infections under several alternative scenarios from (A) Jan 1,2020 to Mar 15,2020, and (B) rom Jan 1, 2020 to July 15, 2020. Data are presented as the median (solid line) and IQR (shading) of estimates (200 simulations). “No Policy” denotes the scenario in which no anti-contagion policies have been put in place. “Travel Restriction” denotes the case where only intercity travel bans were implemented after January 23. “Lockdown” denotes the scenario in which only city lockdown was implemented after January 23. “Testing & Treatment” denotes the scenario in which the detection rates of infections were fixed at the levels before January 23. “Simulation” denotes the baseline scenario in which all three policies have been implemented from January 23 to March 15.

A direct comparison across three groups of control methods was presented in Table 2 and Figs 7 and 8. We found that drastic suppression measures, such as city lockdowns, were most effective in the short run: in the counterfactual scenarios had we lifted city lockdowns after January 23, the cumulative number of infections by February 29 would be 648% of the reality. Comparatively, keeping the detection rate and transmission parameters at the baseline level (before January 23) produced additional 69% infections. Restoring intercity travel flows to the 2019 level would lead to a threefold growth in infected cases out of Wuhan but had limited effects on Wuhan. The three containment measures had strong complementary effects: lifting all three interventions, the number of cases would have been 65-fold higher by February 29, quite close to the estimates from [12].

**Table 2:** Alternative scenario lifting each control method.

	No Control Method	No Travel Ban	No Lockdown	No Testing Treatment
**Panel A: National**				
*Until 7.15*				
No. of cases	1.22×10^9^	3.84×10^5^	2.54×10^6^	1.14×10^9^
simulated cases/actual case	3274.93	1.03	6.81	3069.81
*Until 3.15*				
No. of cases	1.72×10^8^	3.84×10^5^	2.52×10^6^	2.15×10^6^
simulated cases/actual case	462.62	1.03	6.78	5.77
*Until 2.29*				
No. of cases	2.41×10^7^	3.84×10^5^	2.41×10^6^	6.28×10^5^
simulated cases/actual case	64.70	1.03	6.48	1.69
**Panel B: Hubei**				
*Until 7.15*				
No. of cases	5.99×10^7^	3.47×10^5^	2.50×10^6^	2.21×10^7^
simulated cases/actual case	168.81	1.00	7.03	62.17
*Until 3.15*				
No. of cases	4.04×10^7^	3.47×10^5^	2.48×10^6^	5.48×10^5^
simulated cases/actual case	113.89	1.00	6.99	1.54
*Until 2.29*				
No. of cases	1.40×10^7^	3.46×10^5^	2.37×10^6^	4.66×10^5^
simulated cases/actual case	39.36	1.00	6.69	1.31
**Panel C: Other City**				
*Until 7.15*				
No. of cases	1.16×10^9^	3.77×10^4^	4.13×10^4^	1.12×10^9^
simulated cases/actual case	67139.22	2.19	2.39	64909.19
*Until 3.15*				
No. of cases	1.32×10^8^	3.77×10^4^	4.09×10^4^	1.60×10^6^
simulated cases/actual case	7638.59	2.18	2.37	92.81
*Until 2.29*				
No. of cases	1.01×10^7^	3.74×10^4^	3.88×10^4^	1.62×10^5^
simulated cases/actual case	589.17	2.18	2.26	9.46

This table shows alternative counterfactual scenarios lifting each one of the three control measures (lockdown, intercity travel ban and improved testing efficiency) and all three of them together (no control measure). Column (a) shows scenario without any control measure in place since January 23. Column (b), (c) and (d) present counterfactual number of infectious cases under scenarios that lift intercity travel restriction, lockdown and testing and treatment measures, respectively. Results in this table are the mean value derived from 200 simulations.

However, as we extend the time horizon of the analysis, the cumulative effects of different control measures reversed. City lockdown by itself was not sufficient to bring the spread under control: as shown in Table 3, in a counterfactual scenario with both city lockdowns and travel bans in place, leaving detection rate at 30% would predict 1.4×10^8^ (95% credible interval (CI): 6.6×10^7^-3.2×10^8^) cases by July 15, almost 1,650 times the actual infections in reality, compared to 343 times when we keep the detection rate at 70%. On the contrary, with efficient testing, tracing and treatment, we could afford to relax restrictions on both within-city and inter-city mobility. If we manage a detection rate at 70%, restoring within and intercity city mobility to last year level would only lead to a 6.2-fold growth in infected cases. We also explore the spatial distribution of infections under different scenarios.

**Table 3:** Alternative scenario with different testing efficiency.

	30%	50%	70%	90%
**Panel A: Until 3.15**				
*Scenario 1: No lockdown ever*				
No. of cases	1.55×10^7^	4.28×10^6^	2.58×10^6^	2.05×10^6^
simulated cases/actual cases	41.5	11.5	6.9	5.5
*Scenario 2: Lockdown since Jan 23*				
No. of cases	6.28×10^5^	4.70×10^5^	4.40×10^5^	4.27×10^5^
simulated cases/actual cases	1.74	1.30	1.22	1.18
**Panel B: Until 7.15**				
*Scenario 1: No lockdown ever*				
No. of cases	6.95×10^8^	7.72×10^6^	2.68×10^6^	2.07×10^6^
simulated cases/actual cases	1866.9	19.4	7.2	5.6
*Scenario 2: Lockdown since Jan 23*				
Cases	2.22×10^8^	5.01×10^5^	4.51×10^5^	4.41×10^5^
simulated cases/actual cases	595.14	1.38	1.22	1.18
*Scenario 3: Lockdown from Jan 23 to Mar 15*, *no lockdown after Mar 15*				
No. of cases	2.89×10^8^	4.97×10^5^	4.40×10^5^	4.27×10^5^
simulated cases/actual cases	799.59	1.38	1.22	1.18

This table presents alternative counterfactual scenarios with different detection rate after March 15. We let the detection rate to increase from the initial level before January 23, gradually to the designated level from January 23 to March 15. We simulate different lockdown scenarios and set the inner city mobility to follow the level on the same lunar calendar date in 2019 in the counterfactual case of no lockdown. The three scenarios are (1) No lockdown ever from January 10 to July 15; (2) Lockdown from January 10 to July 15; (3) Lockdown from January 10 to March 15, no lockdown from March 15 to July 15. Results in this table are the mean value derived from 200 simulations. After March 15 when the daily mobility data were no longer available, we use the average migration and inner-city flow intensity from March 9 to March 15 (one week before March 15) as a proxy.

## Supporting information

S1 Fig Prediction from March 16 to July 15. These graphs compare the daily official reported number of cases to the simulated ones from 200 simulations using the fully-estimated model from March 15,2020 to July 15,2020. Graph (a) is daily infection case and (b) is cumulative case. The orange x deontes the number of daily reported cases. The blue box and whiskers show the median, interquartile range(IQR), and 1.5IQR derived from 200 simulations using the fitting model with parameters estimated from Table 1. *R*^2^ = 0.86-0.97.
(TIF)

S2 Fig Alternative scenarios with difference testing rate. This figure plots the trends in observed and counterfactual number of cumulative infections under alternative infection detection rates from Jan 1,2020 to July 15,2020. Data are presented as the median (solid line) and IQR (shading) of estimates (200 simulations). In the scenario presented in the upper panel, we keep the other two policies (lockdown and intercity travel ban) in place, and adjust the detection rate of infections to be 30%, 50%, 70% and 90% respectively. In the lower panel, we drop the lockdown and travel ban policies. The other parameters such transmission rate *β* and infectious period 1/*γ* are the same as in the baseline.
(TIF)

S3 Fig Alternative scenarios with different detection rate: Keep lockdown. This figure plots the trends in observed and counterfactual number of cumulative infections under alternative infection detection rates from Jan 1,2020 to Mar 15,2020. Data are presented as the median (solid line) and IQR (shading) of estimates (200 simulations). In the alternative scenarios, we keep the other two policies (lockdown and intercity travel ban) in place, and adjust the detection rate of infections to be 30%, 50%, 70% and 90% respectively. The other parameters such transmission rate *β* and infectious period 1/*γ* are the same as in the baseline.
(TIF)

S4 Fig Simulation with different initial value. This graph plot simulation by using different initial value. The data is better fitted when initial value equal to 3000 (MAE = 363) than initial value equal to other value. (A *MAE*_*seed* = 1000_ = 848, B *MAE*_*seed* = 2000_ = 489, C *MAE*_*seed* = 2500_ = 373, D *MAE*_*seed* = 3000_ = 354, E*MAE*_*seed* = 3500_ = 438, F*MAE*_*seed* = 4000_ = 554.
(TIF)

S5 Fig Delay event study. t0 is January 31,2020, We observed the change of delay between pre-13 days (1.18) and post-15 days in which hospital registration data is available. According to the graph, we divided the delay into two periods (before and after February 1).
(TIF)

S6 Fig Delay distribution. Distribution of interval between the day admitted by hospital and confirmation day for cases confirmed before(A) and after (B) February 1,2020. The data prior to February 1 were fitted with a Gamma distribution(a = 3.55, b = 1.22, LL = 1669.71) and data were fitted with a Gamma distribution (a = 3.86, b = 0.78, LL = 522.82 after 2.1) after February 1,2020.
(TIF)

S7 Fig Calibration of intercity mobility data from Baidu. This graph shows the value range of mobility data from mobile phones divided by data from Baidu. Mobility data is from [4], they provide the sum of outflow population from Wuhan in January 1 to January 24.
(TIF)

S8 Fig Global sensitivity analysis for interested parameter. This graph presents the sensitivity indices for our model. Following [15], we use the SAFE toolbox to conduct the Global Sensitivity Analysis (GSA) [16]. The parameters included in this analysis were the reported rate r_*i*,*i*=1,2,3_ in the first four periods.
(TIF)

S9 Fig Comparison of the best and worst result of the GSA, the optimized model and the real data. Following [15], we use the SAFE toolbox to conduct the Global Sensitivity Analysis (GSA). The parameters included in this analysis were the reported rate r_*i*,*i*=1,2,3_ in the first four periods.
(TIF)

## References

[1] López L, Rodó X. The end of social confinement and COVID-19 re-emergence risk. Nature Human Behaviour. 2020;4(7):746–755. doi: 10.1038/s41562-020-0908-8 32572175

[2] López L, Rodo X. A modified SEIR model to predict the COVID-19 outbreak in Spain and Italy: simulating control scenarios and multi-scale epidemics. Results in Physics. 2021;21:103746. doi: 10.1016/j.rinp.2020.103746 33391984 PMC7759445

[3] Bohk-Ewald C, Dudel C, Myrskylä M. A demographic scaling model for estimating the total number of COVID-19 infections. arXiv preprint arXiv:200412836. 2020;. 10.1093/ije/dyaa198 PMC7799106 33349859

[4] Jia JS, Lu X, Yuan Y, Xu G, Jia J, Christakis NA. Population flow drives spatio-temporal distribution of COVID-19 in China. Nature. 2020; p. 1–5. 32349120 10.1038/s41586-020-2284-y

[5] Li R, Pei S, Chen B, Song Y, Zhang T, Yang W, et al. Substantial undocumented infection facilitates the rapid dissemination of novel coronavirus (SARS-CoV-2). Science. 2020;368(6490):489–493. doi: 10.1126/science.abb3221 32179701 PMC7164387

[6] López L, Fernández M, Gómez A, Giovanini L. An influenza epidemic model with dynamic social networks of agents with individual behaviour. Ecological Complexity. 2020;41:100810. doi: 10.1016/j.ecocom.2020.100810

[7] Chen S, Zhang Z, Yang J, Wang J, Zhai X, Bärnighausen T, et al. Fangcang shelter hospitals: a novel concept for responding to public health emergencies. The Lancet. 2020;. doi: 10.1016/S0140-6736(20)30744-3 32247320 PMC7270591

[8] Shen M, Peng Z, Xiao Y, Zhang L. Modelling the epidemic trend of the 2019 novel coronavirus outbreak in China. BioRxiv. 2020;. 10.1016/j.xinn.2020.100048 PMC7831648 33521762

[9] Read JM, Bridgen JR, Cummings DA, Ho A, Jewell CP. Novel coronavirus 2019-nCoV: early estimation of epidemiological parameters and epidemic predictions. MedRxiv. 2020;. 10.1098/rstb.2020.0265 PMC8165596 34053269

[10] Cao Z, Zhang Q, Lu X, Pfeiffer D, Jia Z, Song H, et al. Estimating the effective reproduction number of the 2019-nCoV in China. MedRxiv. 2020;.

[11] Tang B, Wang X, Li Q, Bragazzi NL, Tang S, Xiao Y, et al. Estimation of the transmission risk of the 2019-nCoV and its implication for public health interventions. Journal of clinical medicine. 2020;9(2):462. doi: 10.3390/jcm9020462 32046137 PMC7074281

[12] Lai S, Ruktanonchai N, Zhou L, Prosper O, Luo W, Floyd J, et al. Effect of non-pharmaceutical interventions to contain COVID-19 in China. Nature. 2020;. doi: 10.1038/s41586-020-2293-x 32365354 PMC7116778

[13] Ku CC, Ng TC, Lin HH. Epidemiological benchmarks of the COVID-19 outbreak control in China after Wuhan’s lockdown: a modelling study with an empirical approach. Available at SSRN 3544127. 2020;.

[14] Chinazzi M, Davis JT, Ajelli M, Gioannini C, Litvinova M, Merler S, et al. The effect of travel restrictions on the spread of the 2019 novel coronavirus (COVID-19) outbreak. Science. 2020;368(6489):395–400. doi: 10.1126/science.aba9757 32144116 PMC7164386

[15] López L, Izquierdo A, Manzoli D, Beldoménico P, Giovanini L. A myiasis model for Philornis torquans (Diptera: Muscidae) and Pitangus sulphuratus (Passeriformes: Tyrannidae). Ecological Modelling. 2016;328:62–71. doi: 10.1016/j.ecolmodel.2016.02.001

[16] Pianosi F, Sarrazin F, Wagener T. A Matlab toolbox for global sensitivity analysis. Environmental Modelling & Software. 2015;70:80–85. doi: 10.1016/j.envsoft.2015.04.009

