# Supplementary figures and images for: Control strategies against COVID-19 in China: Significance of effective testing in the long run
Source: PLoS One. 2021 Jul 9;16(7):e0253901. doi: 10.1371/journal.pone.0253901 (PMC8270456; doi:10.1371/journal.pone.0253901)

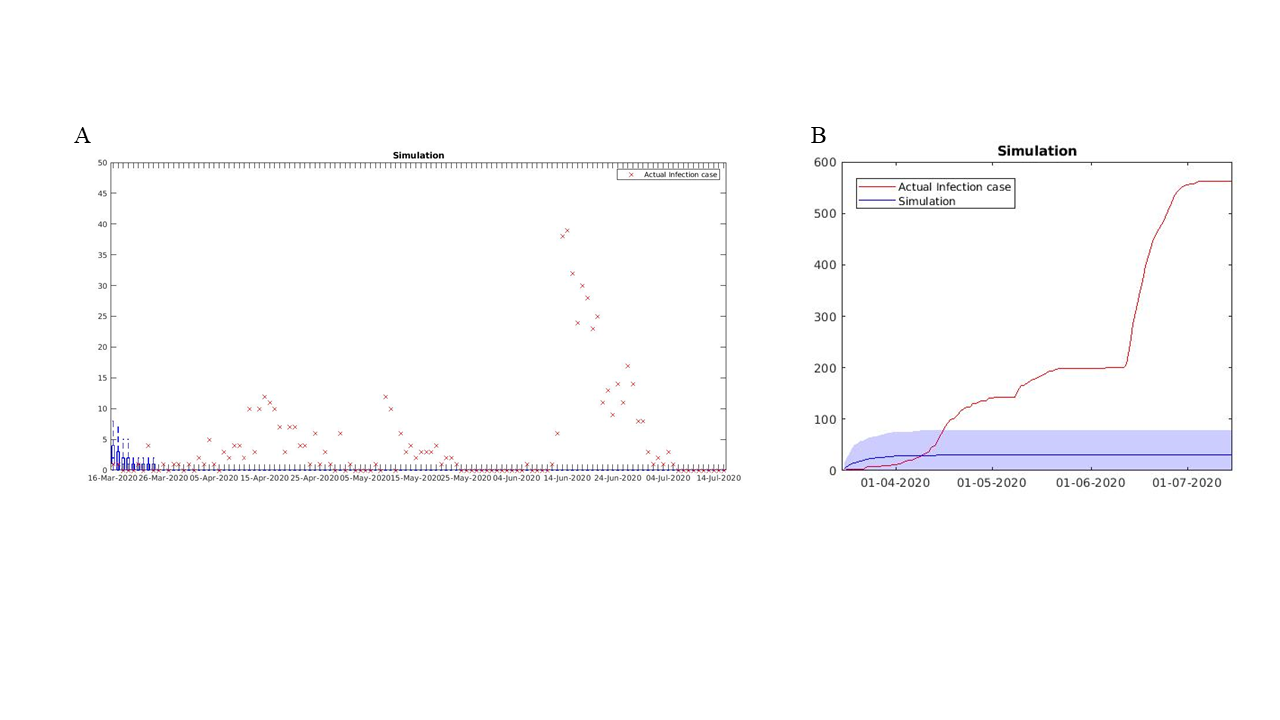

Supplement: S1 Fig — These graphs compare the daily official reported number of cases to the simulated ones from 200 simulations using the fully-estimated model from March 15,2020 to July 15,2020. Graph (a) is daily infection case and (b) is cumulative case. The orange x deontes the number of daily reported cases. The blue box and whiskers show the median, interquartile range(IQR), and 1.5IQR derived from 200 simulations using the fitting model with parameters estimated from Table 1. R2 = 0.86-0.97. (TIF) [file pone.0253901.s001.tif]

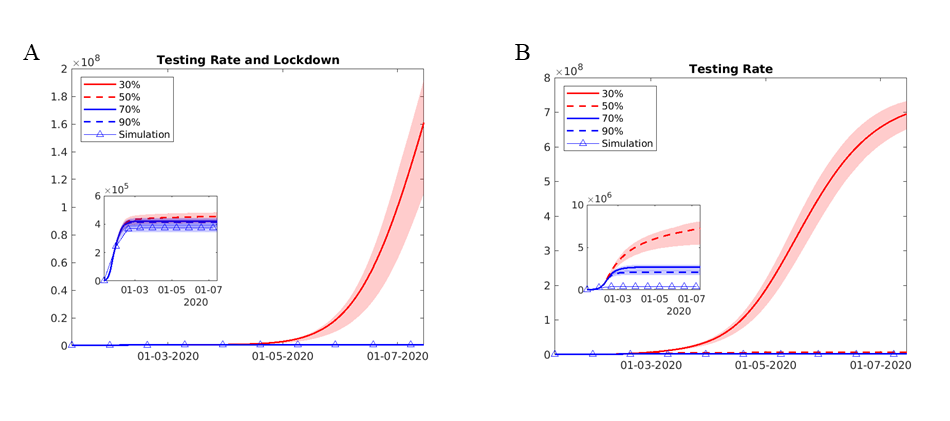

Supplement: S2 Fig — This figure plots the trends in observed and counterfactual number of cumulative infections under alternative infection detection rates from Jan 1,2020 to July 15,2020. Data are presented as the median (solid line) and IQR (shading) of estimates (200 simulations). In the scenario presented in the upper panel, we keep the other two policies (lockdown and intercity travel ban) in place, and adjust the detection rate of infections to be 30%, 50%, 70% and 90% respectively. In the lower panel, we drop the lockdown and travel ban policies. The other parameters such transmission rate β and infectious period 1/γ are the same as in the baseline. (TIF) [file pone.0253901.s002.tif]

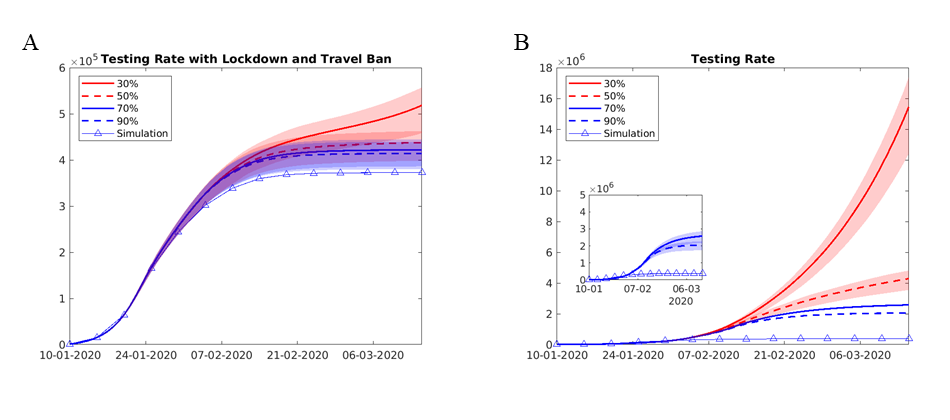

Supplement: S3 Fig — This figure plots the trends in observed and counterfactual number of cumulative infections under alternative infection detection rates from Jan 1,2020 to Mar 15,2020. Data are presented as the median (solid line) and IQR (shading) of estimates (200 simulations). In the alternative scenarios, we keep the other two policies (lockdown and intercity travel ban) in place, and adjust the detection rate of infections to be 30%, 50%, 70% and 90% respectively. The other parameters such transmission rate β and infectious period 1/γ are the same as in the baseline. (TIF) [file pone.0253901.s003.tif]

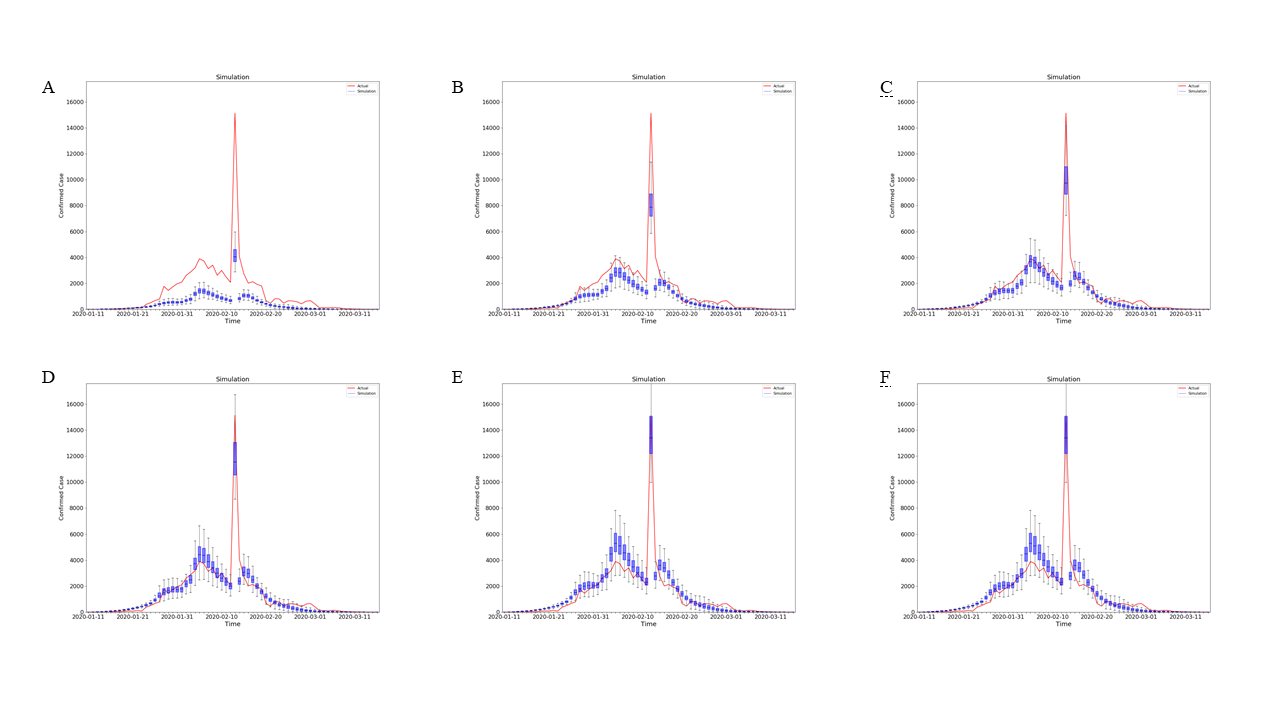

Supplement: S4 Fig — This graph plot simulation by using different initial value. The data is better fitted when initial value equal to 3000 (MAE = 363) than initial value equal to other value. (A MAEseed = 1000 = 848, B MAEseed = 2000 = 489, C MAEseed = 2500 = 373, D MAEseed = 3000 = 354, EMAEseed = 3500 = 438, FMAEseed = 4000 = 554. (TIF) [file pone.0253901.s004.tif]

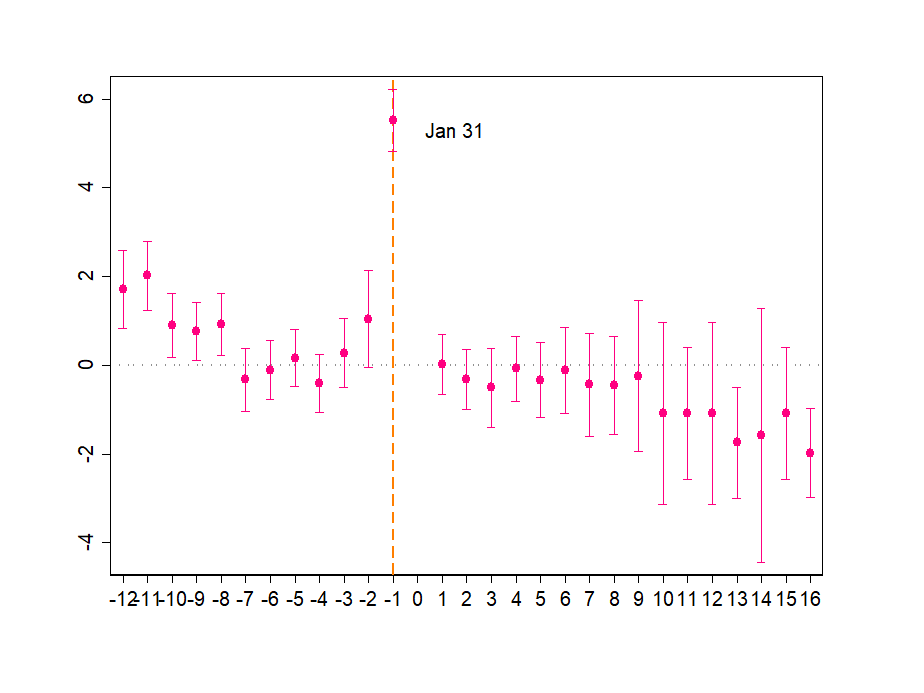

Supplement: S5 Fig — t0 is January 31,2020, We observed the change of delay between pre-13 days (1.18) and post-15 days in which hospital registration data is available. According to the graph, we divided the delay into two periods (before and after February 1). (TIF) [file pone.0253901.s005.tif]

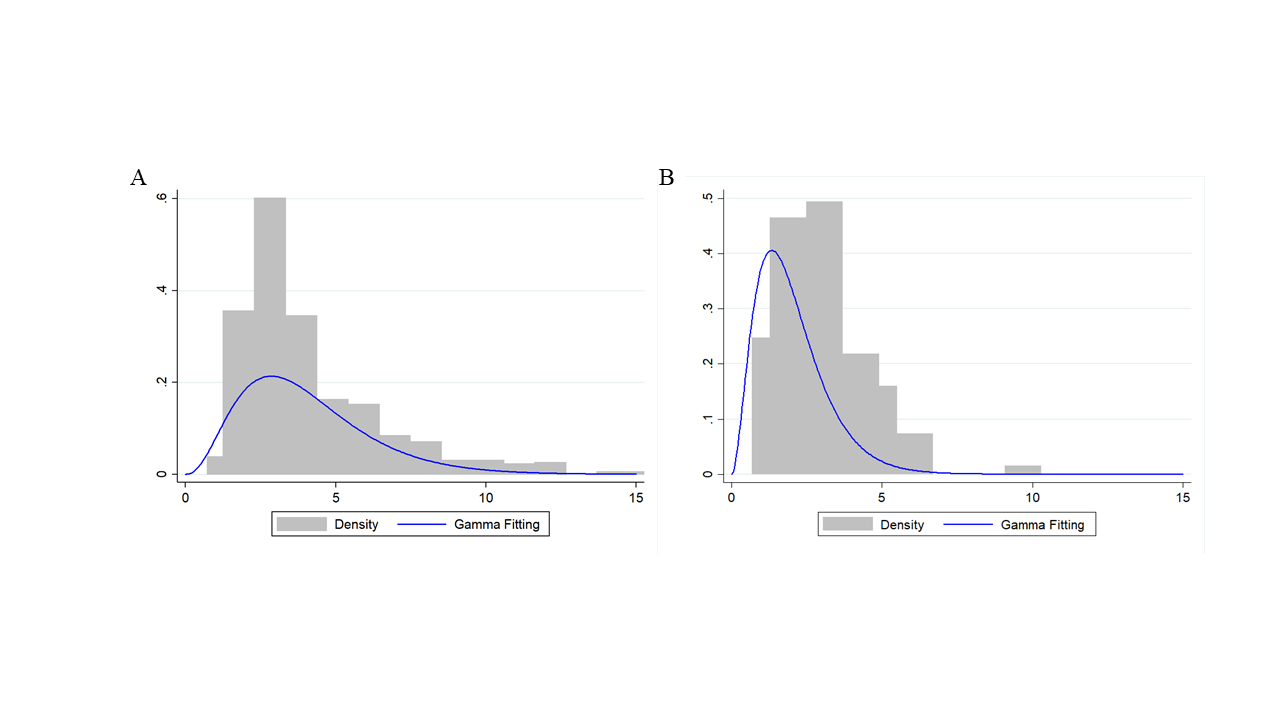

Supplement: S6 Fig — Distribution of interval between the day admitted by hospital and confirmation day for cases confirmed before(A) and after (B) February 1,2020. The data prior to February 1 were fitted with a Gamma distribution(a = 3.55, b = 1.22, LL = 1669.71) and data were fitted with a Gamma distribution (a = 3.86, b = 0.78, LL = 522.82 after 2.1) after February 1,2020. (TIF) [file pone.0253901.s006.tif]

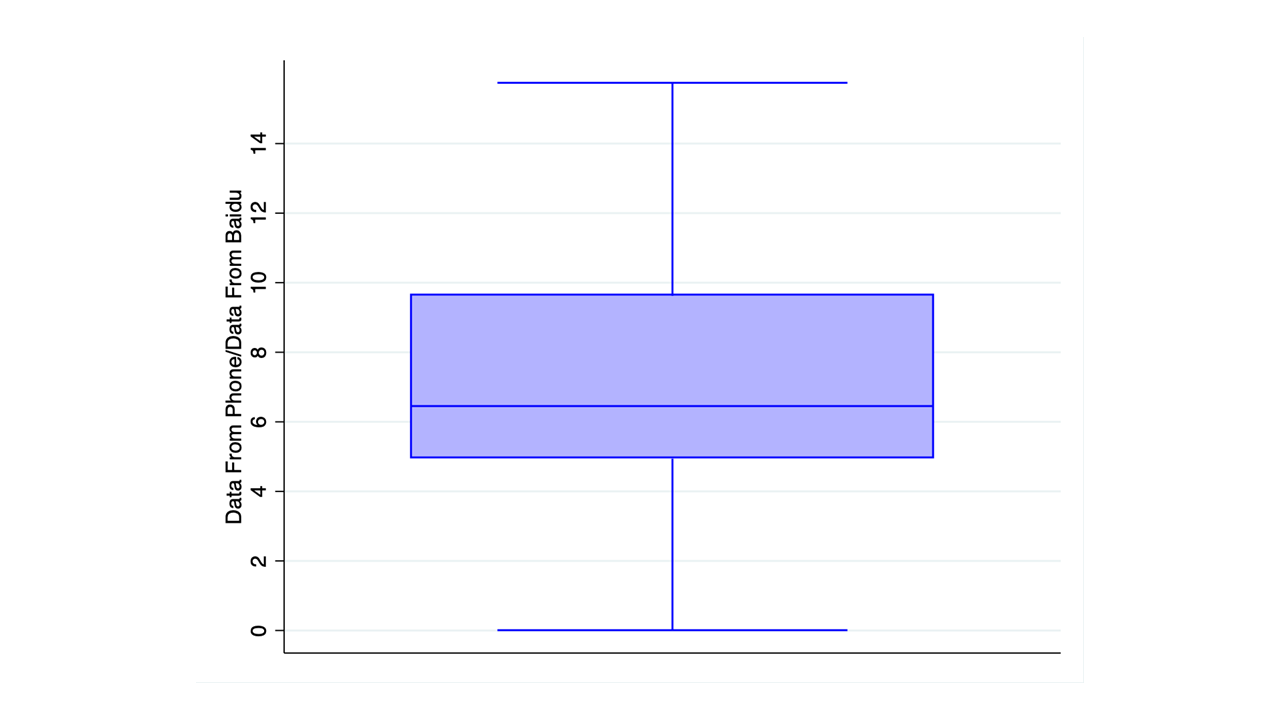

Supplement: S7 Fig — This graph shows the value range of mobility data from mobile phones divided by data from Baidu. Mobility data is from [4], they provide the sum of outflow population from Wuhan in January 1 to January 24. (TIF) [file pone.0253901.s007.tif]

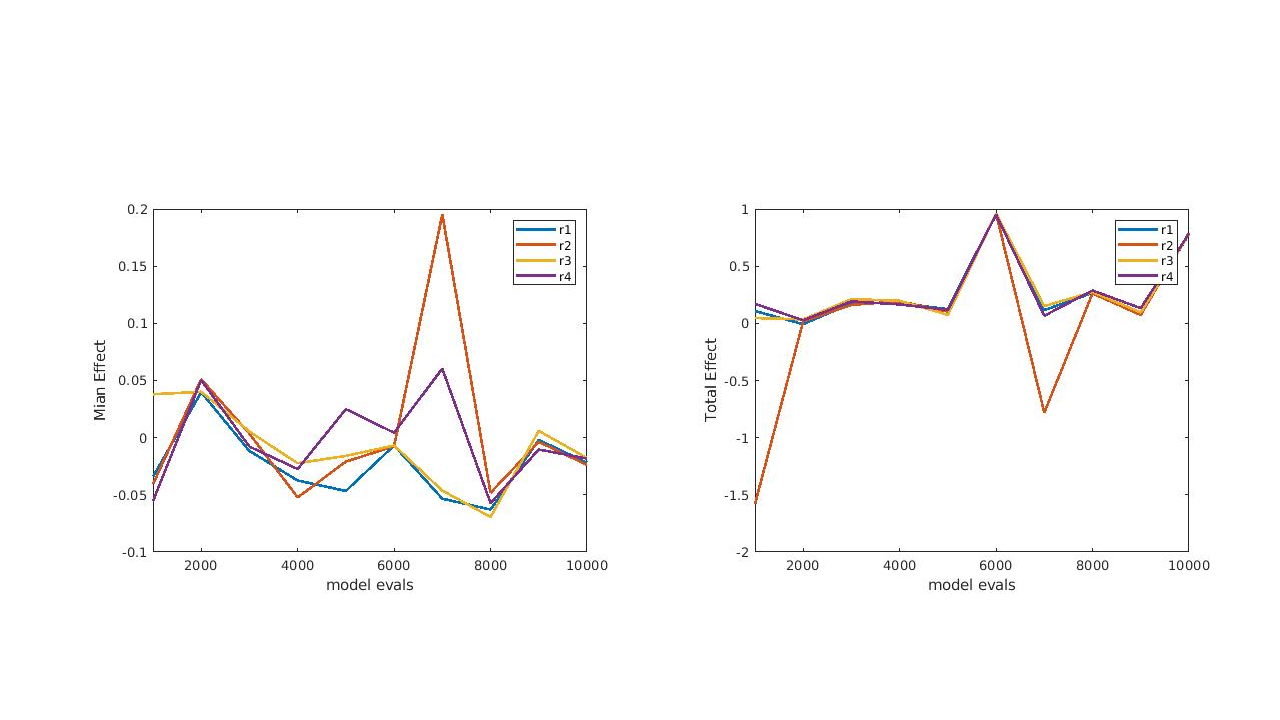

Supplement: S8 Fig — This graph presents the sensitivity indices for our model. Following [15], we use the SAFE toolbox to conduct the Global Sensitivity Analysis (GSA) [16]. The parameters included in this analysis were the reported rate ri,i=1,2,3 in the first four periods. (TIF) [file pone.0253901.s008.tif]

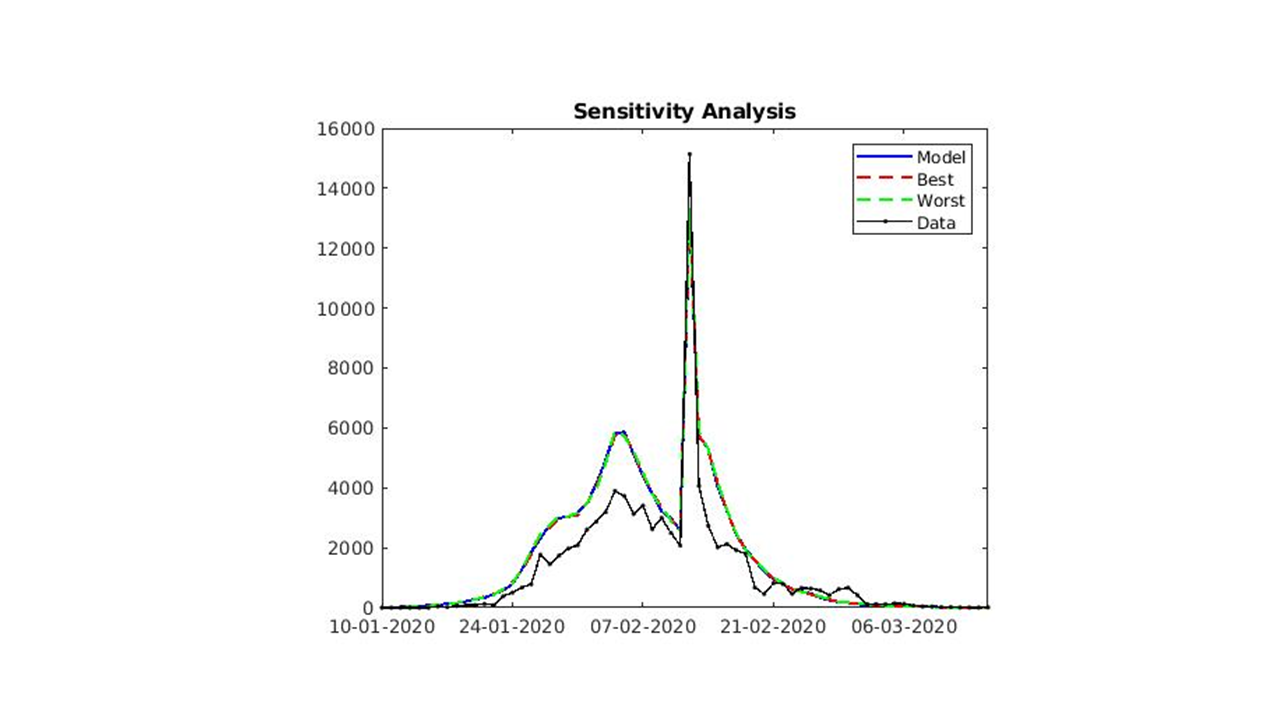

Supplement: S9 Fig — Following [15], we use the SAFE toolbox to conduct the Global Sensitivity Analysis (GSA). The parameters included in this analysis were the reported rate ri,i=1,2,3 in the first four periods. (TIF) [file pone.0253901.s009.tif]
